# Transcriptome Analysis of Larval Segment Formation and Secondary Loss in the Echiuran Worm *Urechis unicinctus*

**DOI:** 10.3390/ijms20081806

**Published:** 2019-04-12

**Authors:** Xitan Hou, Maokai Wei, Qi Li, Tingting Zhang, Di Zhou, Dexu Kong, Yueyang Xie, Zhenkui Qin, Zhifeng Zhang

**Affiliations:** College of Marine Life Sciences, Ocean University of China, Qingdao 266003, China; houxitan@126.com (X.H.); maokaiwei1992@163.com (M.W.); Vickyqi1015@163.com (Q.L.); 15615426077@163.com (T.Z.); zhoudi0628@126.com (D.Z.); 18254308601@163.com (D.K.); yueyangyouxiang@126.com (Y.X.)

**Keywords:** *Urechis unicinctus*, Echiura, transcriptome, segmentation, Hedgehog pathway

## Abstract

The larval segment formation and secondary loss in echiurans is a special phenomenon, which is considered to be one of the important characteristics in the evolutionary relationship between the Echiura and Annelida. To better understand the molecular mechanism of this phenomenon, we revealed the larval transcriptome profile of the echiuran worm *Urechis unicinctus* using RNA-Seq technology. Twelve cDNA libraries of *U. unicinctus* larvae, late-trochophore (LT), early-segmentation larva (ES), segmentation larva (SL), and worm-shaped larva (WL) were constructed. Totally 243,381 unigenes were assembled with an average length of 1125 bp and N50 of 1836 bp, and 149,488 unigenes (61.42%) were annotated. We obtained 70,517 differentially expressed genes (DEGs) by pairwise comparison of the larval transcriptome data at different developmental stages and clustered them into 20 gene expression profiles using STEM software. Based on the typical profiles during the larval segment formation and secondary loss, eight signaling pathways were enriched, and five of which, mTOR, PI3K-AKT, TGF-β, MAPK, and Dorso-ventral axis formation signaling pathway, were proposed for the first time to be involved in the segment formation. Furthermore, we identified 119 unigenes related to the segment formation of annelids, arthropods, and chordates, in which 101 genes were identified in *Drosophila* and annelids. The function of most segment polarity gene homologs (*hedgehog*, *wingless*, *engrailed*, etc.) was conserved in echiurans, annelids, and arthropods based on their expression profiles, while the gap and pair-rule gene homologs were not. Finally, we verified that strong positive signals of Hedgehog were indeed located on the boundary of larval segments using immunofluorescence. Data in this study provide molecular evidence for the understanding of larval segment development in echiurans and may serve as a blueprint for segmented ancestors in future research.

## 1. Introduction

Echiurans are a group of marine benthic invertebrates including approximately 230 species, which all inhabit marine environments from the intertidal zone to thousands of meters in the deep sea [1,2,3], such as *Bonellia viridis* in coastal sediment and *Hydroides elegans* in the deep sea. Academically, it has been a controversial issue whether the echiurans belong to Annelida or a separate phylum Echiura [4]. One view is that echiurans are a subtaxon of annelid based on their corresponding morphological information, such as the ultrastructure of cuticle and cilium [5], and ladder-like nervous system [6], as well as recent molecular phylogenetic analyses [1,7,8,9]. However, other researchers considered that echiurans should not be attributed to annelids due to the presence of a few setae, the lack of eyes or other distinct sense organs, or especially the lack of segmentation in adult echiurans [10]. They should be considered to have diverged from annelids [11].

Segmentation appears in several animal phyla, including annelids, arthropods, chordates, etc. [12]. The body segments in annelids and arthropods are referred to be the typical segmentation [13]. In these animals, the segmentation is not only distinguishable in the external morphology but also in the internal systems, such as the nervous [14], circulatory [13], excretion [13], and reproductive [15] systems. Annelids are the most conspicuously segmented animals as most of the trunk is formed from identical anatomical units [13]. During the body segment formation in annelids, the grooves of the body wall produce first, and then the muscle groups are differentiated and segmented [13]. Therefore, both ectoderm and mesoderm (muscle) in annelids are involved in the formation of the body segments which are maintained throughout its lifetime. In echiurans, the segments are maintained only at the larval stage and formed by invagination of the body wall consisting of ectoderm only [16]. Due to the temporary segments and no segmented muscle in the segment regions, thus we defined them as the larval segments in echiurans. Based on the obvious difference between the larval segmentation in echiurans and the typical segmentation in annelids, it is of great significance to explore the molecular mechanism of larval segment formation in echiurans.

So far, two important mechanisms of segment formation have been proposed, the “segmentation clocks” in vertebrates [17,18] and the “segmentation cascade” in arthropods [19,20]. The segmentation clock is an autonomous periodic mechanism, which is controlled by a cyclic signaling network involving the notch, FGF, and Wnt pathways [21]. The segmentation cascade is supported by the related segment gene cascades, which has been well understood in the fruit fly *Drosophila melanogaster*, such as gap genes (*hb*, *gt*, *kr*, etc.), pair-rule genes (*ftz*, *eve*, *odd*, etc.), and segment polarity genes (*engrailed*, *hedgehog, wingless*, etc.) [13,22]. Currently, most of the research on segment formation in annelids follows the segmentation cascade mechanism [19,23,24,25,26,27].

**Figure 1:**
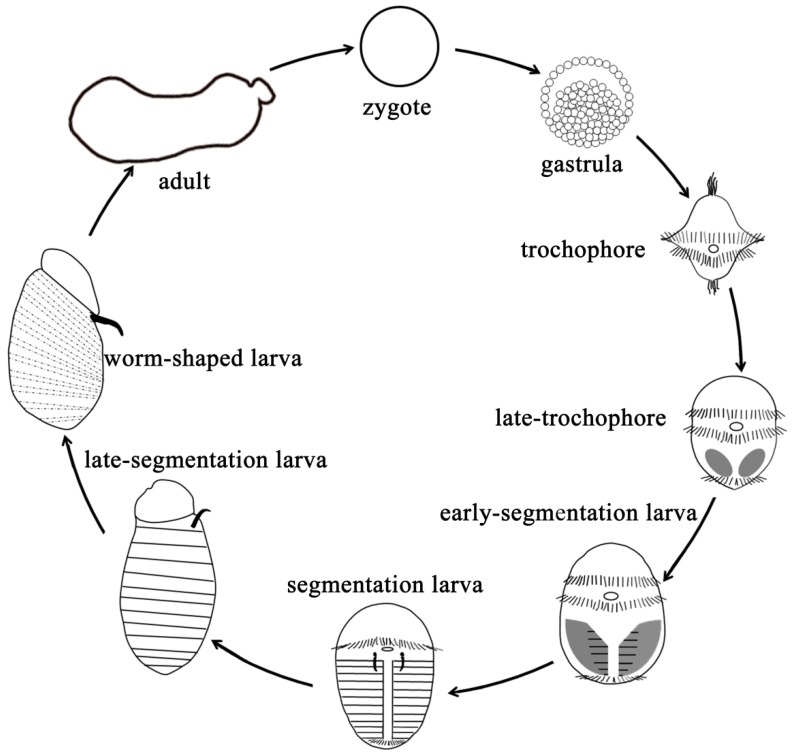
The life cycle of *Urechis unicinctus*. The gastrula hatches and develops into a swimming larva, the trochophore, in which a prototroch separates the larva into the episphere (upper hemisphere) and the hyposphere (lower hemisphere) to form the head and the trunk, respectively. The segments occur firstly in the early-segmentation larva and then maintain through the late-segmentation larva. The secondary loss of the segments appears in the worm-shaped larva whose inhabit mode changes from free-living into burrowing in the sediment. A body plan without segments is kept in adult *U. unicinctus*.

To obtain the related genes of larval segment formation in echiurans, we employed the spoon worm *Urechis unicinctus*, an important commercial echiuran, which mainly inhabits in the coastal sediment of China, Korea, Japan, and Russia [28,29]. The transcriptomic data were obtained from *U. unicinctus* larvae during segment formation and secondary loss (Figure 1), including late-trochophore (LT), early-segmentation larva (ES), segmentation larva (SL), and worm-shaped larva (WL), using the next-generation RNA sequencing technique. We analyzed the transcriptomic characteristics and identified the segment-related genes and pathways. Furthermore, a spatial location of the Hedgehog was verified in *U. unicinctus* larvae using an immunofluorescence technique. This study provides basic data to study the evolution of segment formation mechanism in animals.

## 2. Results

### 2.1. Illumina Sequencing, De Novo Assembly and Functional Annotation

To obtain an overview of the *U. unicinctus* larval transcriptome, twelve cDNA libraries were constructed from the larvae of four developmental stages, late-trochophore (LT), early-segmentation larva (ES), segmentation larva (SL), and worm-shaped larva (WL). A total of 609.53 Mb raw reads were generated and 591.58 Mb clean reads were obtained (Table 1). These clean reads were then *de novo* assembled by Trinity software and generated 243,381 unigenes (*gene* and *unigene* used hereinafter all represent *unigene*) with an average length of 1125 bp and N50 of 1836 bp (Table 2). The PCA analysis showed that three biological replicates of each developmental stage segregated together, respectively, and LT and ES samples tended to cluster as well (Figure S1), which indicated the reliability of the data. All RNA-Seq data had been submitted to the NCBI Sequence Read Archive under the accession number of SRP156975.

All the unigenes were aligned against seven public databases with a cutoff E-value < 1 × 10^−5^, including the NCBI non-redundant protein/nucleotide database (NR and NT), the Kyoto Encyclopedia of Genes and Genomes Ortholog (KO), the Swiss-Prot protein database (SwissPort), the Protein family (Pfam), the Gene Ontology (GO), and the euKaryotic Ortholog Groups (KOG) databases. Totally 149,488 unigenes (61.42% of all the 243,381 unigenes) were annotated, and 7,704 (3.16%) unigenes were simultaneously annotated by the seven databases (Table 3).

**Figure 2:**
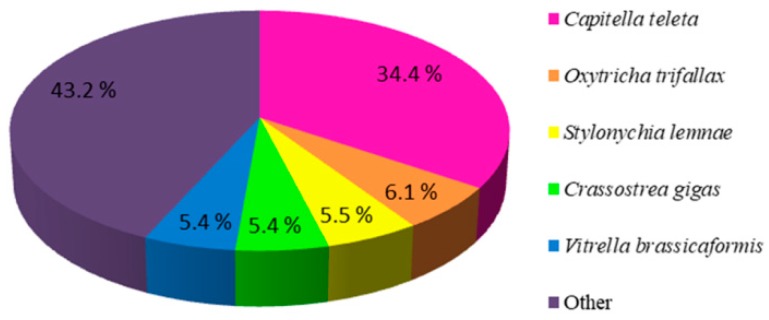
Species distribution of the unigenes from *U. unicinctus* larval transcriptome with blast in NR database.

The distribution of the best blast hits over species showed that the top five matched species of the unigene numbers were *Capitella teleta* (34.4%), *Oxytricha trifallax* (6.1%), *Stylonychia lemnae* (5.5%), *Crassostrea gigas* (5.4%), and *Vitrella brassicaformis* (5.4%) (Figure 2).

GO analysis showed that a total of 111,479 unigenes (45.8% of the total annotated sequences) were assigned at least one GO term. These unigenes were sorted into 56 level-2 GO terms under three main GO categories: biological processes, molecular functions, and cellular components (Figure S2A). Based on the assigned GO terms, unigenes were found to be highly enriched in “cellular process” (62,603, 56.2%), “metabolic process” (54,080, 48.5%), and “single-organism process” (50,858, 45.6%) under the biological process category; “binding” (60,844, 54.6%), “catalytic activity” (46,129, 41.4%), and “transporter activity” (9605, 8.6%) under the molecular function. In the cellular component category, the top three GO terms were “cell” (32,853, 29.5%), “cell part” (32,844, 29.4%) and “organelle” (22,612, 20.3%).

The KOG database is used to classify orthologous proteins, and the 71,895 unigenes were clustered into 26 categories (Figure S2B). The clusters for “signal transduction mechanisms” (12,663, 17.6%) and “general function prediction only” (12,332, 17.2%) were the top two large terms, followed by “post-translational modification, protein turnover, chaperones” (9285, 12.9%) and “translation, ribosomal structure, and biogenesis” (6059, 8.4%).

KEGG analysis is often used as a powerful tool to identify the biological pathways related to functional unigenes. In this study, a total of 33,027 unigenes were assigned to specific categories in the KEGG database (Figure S2C), and the maximum number of unigenes fell into “signal transduction” (4444, 13.5%), and then “translation” (3474, 10.5%), followed by “transport and catabolism” (2933, 8.9%), “endocrine system” (2881, 6.9%) and “folding, sorting, and degradation” (2046, 6.2%).

### 2.2. Identification, Classification, and Validation of the Differentially Expressed Genes (DEGs)

**Figure 3:**
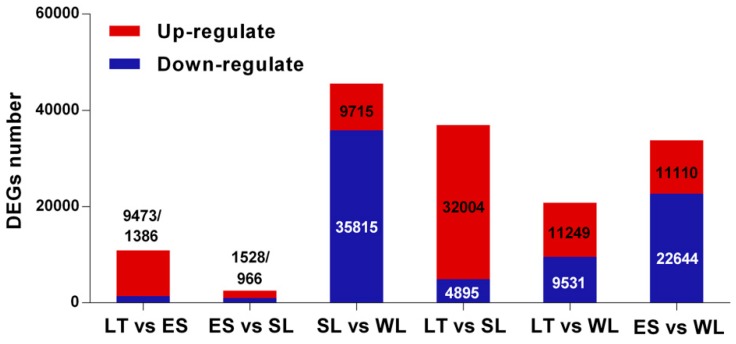
DEGs profiles of *U. unicinctus* larval transcriptome with a pairwise comparison of each time point.

Pairwise comparisons (LT vs. ES, ES vs. SL, SL vs. WL, LT vs. SL, LT vs. WL, and ES vs. WL) were performed to screen the DEGs by using DEGseq with the criteria of FDR ≤ 0.05 and |log2(foldchange)| ≥ 1. A total of 70,517 DEGs were obtained (Figure 3), of which the most were assigned to pairwise comparisons of SL vs. WL, which may be related to *U. unicinctus* larvae experiencing a series of transformations in living habit (from swimming larvae to benthic juveniles) and especially in morphology (disappearance of the body segments and peritroch, shrink of the upper hemisphere, etc.). In contrast, few DEGs were assigned to pairwise comparisons of ES vs. SL. During this period, there are few changes in *U. unicinctus* morphology, except that the body segments are further matured, which is consistent with the actual developmental process.

**Figure 4:**
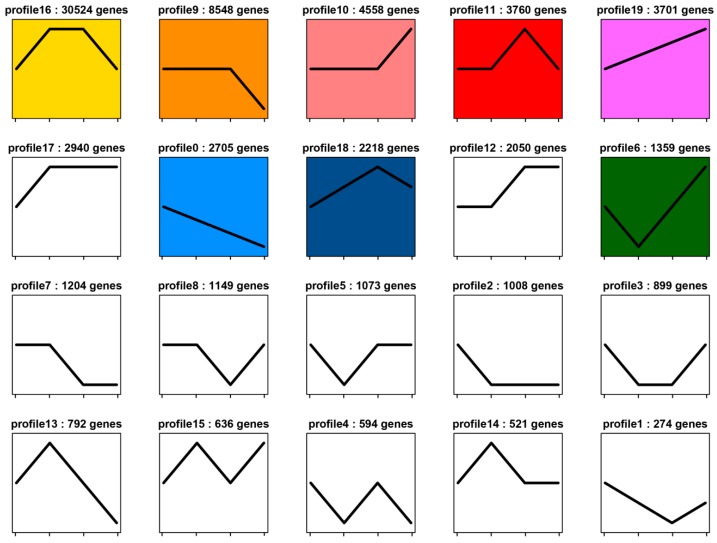
Expression patterns of the DEGs across LT, ES, SL, and WL stages of *U. unicinctus*. The x-axis represents the larval developmental stages, which are LT, ES, SL, and WL from left to right in each profile; the y-axis represents DEGs expression trends.

To get dynamic expression patterns of DEGs during the development process, the STEM software program (Short Time-series Expression Miner, designed for clustering, comparing, and visualizing gene expression data [30]) was utilized to classify all the DEGs according to their abundance changes. Twenty profiles were produced according to their FPKM values, of which eight significant expression profiles (profile 0, profile 6, profile 9, profile 10, profile 11, profile 16, profile 18, and profile 19) were identified, and marked with different background colors (Figure 4). Profile 16 and profile 9 were the two represented patterns as most segment formation-related genes in *U. unicinctus* were assigned to these two profiles.

**Figure 5:**
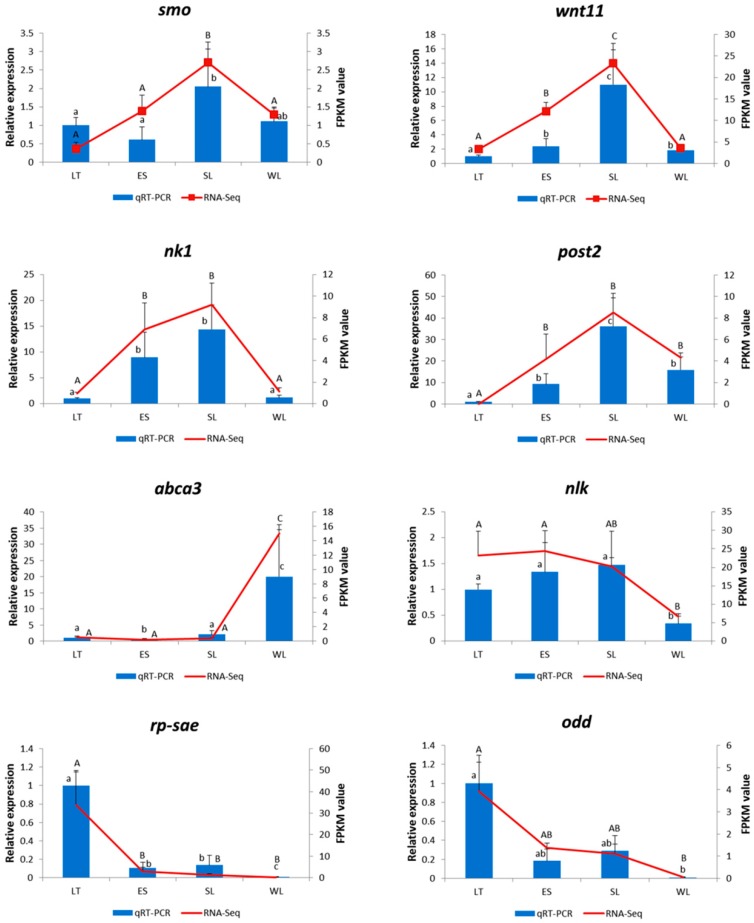
Validation of the RNA-Seq data using qRT-PCR. The blue columns represent the qRT-PCR results; the red lines show the FPKM value. Different characters indicate the significant difference (*p* < 0.05), with the uppercase letter for qRT-PCR and the lowercase for RNA-Seq. *smo* belongs to profile11, *wnt11* and *post2* belong to profile18, *nk1* belongs to profile16, *abca3* belongs to profile10, *nlk* and *odd* belong to profile9 and *rp-sae* belongs to profile2, respectively.

To validate the RNA-Seq results, eight genes (*wnt11*, *smo*, *nk1*, *post2*, *abca3*, *nlk*, *rp-sae*, and *odd*) which represented different gene expression patterns were selected for further confirmation with qRT-PCR. The results showed that the vast majority of the chosen unigenes displayed concordant expression tendencies as in RNA-Seq (Figure 5), confirming that these RNA-Seq data were reliable to quantify gene expression accurately.

### 2.3. Expression Profiles of Genes Related to Segment Formation

**Figure 6:**
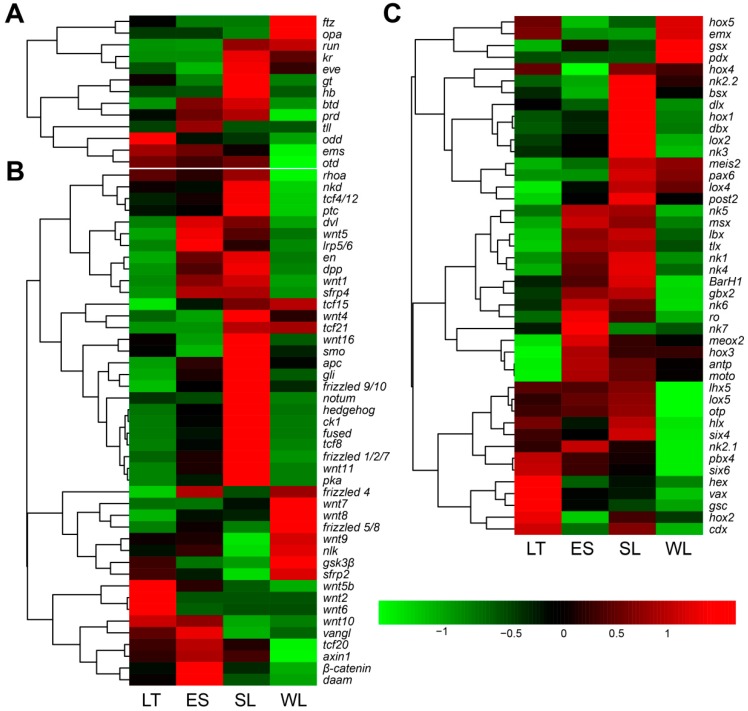
Hierarchical clustering of the segment formation homologous genes in *U. unicinctus* larval transcriptome. (**A**), Gap genes and pair-rule genes; (**B**), segment polarity genes; (**C**), homeotic genes. The clustering indicates similar expression patterns among the genes. The expression levels of genes are presented in the color tape from low (green) to high (red).

In the larval transcriptomes of *U. unicinctus*, a total of 119 unigenes related to the segment formation of annelids, arthropods, and chordates were identified. Out of these genes, 101 genes (Table S1) were known to be involved in the segment formation in *Drosophila* and annelids, including 7 gap genes (*krüppel*, *hunchback*, *giant*, *tailless*, etc.), 6 pair-rule genes (*hairy*, *even-skipped*, *runt*, etc.), 44 segment polarity like genes (*hedgehog*, *wingless*, *engrailed*, etc.) and 44 homeotic genes (*Hox*, *Para-hox*, and *NK* genes). We performed hierarchical clustering of the 101 segment formation-related gene homologs to examine the similarity and diversity of expression profiles (Figure 6). The results showed that all the 7 gap genes and most of the pair-rule genes (5 of 6, except *odd*) were expressed stably (*p* > 0.05) during the progress of segment formation (from LT to ES). However, 13 of 44 segment polarity genes were up-regulated from LT to ES, while down-regulated from SL to WL. One of 10 *Hox* genes (*lox5*), 1 of 3 *ParaHox* genes (*pdx*), and 8 of 22 *NK* genes (*NK1*, *NK4*, *Lbx*, *Msx*, *Tlx*, *NK5*, *Dbx*, and *Vax*) were down-regulated from SL to WL.

### 2.4. Expression Characteristics of Hedgehog (HH) in U. unicinctus Larvae

**Figure 7:**
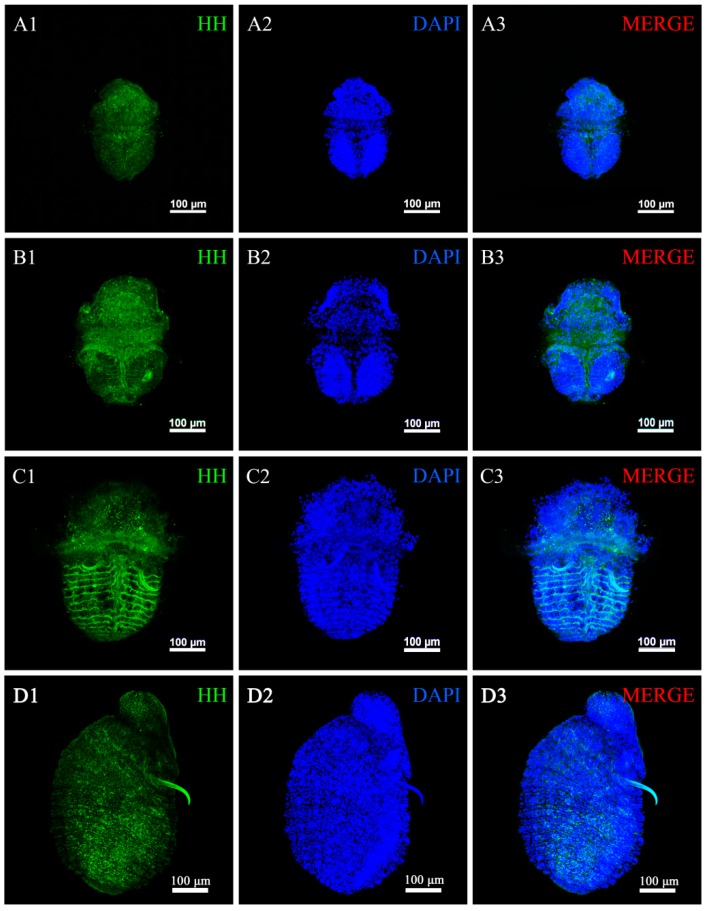
Immunofluorescence assay of HH in *U. unicinctus* larvae during the segment formation and secondary loss. (**A**–**D**) correspond to LT, ES, SL, and WL of *U. unicinctus*, respectively. (**A**–**C**), ventral views; (**D**), lateral view.

The *U. unicinctus* larva transcriptional data showed that *hedgehog* was stable and highly expressed in ES and SL, but the expression level was significantly decreased in WL. To explore the possible role of HH in processes of *U. unicinctus* segment formation and secondarily loss, we detected the expression pattern of HH using immunohistochemistry technology. The results showed that HH positive signals were widely distributed throughout the body (Figure 7). However, the strong HH signals appeared in the boundary of larval segments in the ES and SL (Figure 7B1,C1), and then the HH signals weakened and lost its original expression pattern in the WL, an unsegmented larva (Figure 7D). Negative controls were shown in Figure S3.

## 3. Discussion

### 3.1. Function of Gap and Pair-Rule Genes in U. unicinctus Was Not Consistent with Drosophila during Segmentation

According to the morphological feature in *U. unicinctus* larvae, the visible larval segments occur initially in the developmental process from LT to ES, and are maintained from ES to SL, and then the segments lose secondarily in WL [31]. As seen in hierarchical clustering (Figure 6A) that most of the gap and pair-rule genes (12 of 13) expressed stably (*p* > 0.05) in *U. unicinctus* larvae during the segment formation from LT to ES, except *odd*, implying that the regulation mode of genes involved in the larval segment formation of *U. unicinctus* may not be completely consistent with *Drosophila*. This result is consistent with the pair-rule orthologs examined in the Annelid *Capitella teleta* that none of the pair-rule genes exhibit segmental or pair-rule stripes of expression in the ectoderm or mesoderm [23]. By contrast, 13 of 44 segment polarity genes (*en*, *hh*, *wg*, *gli*, *β-catenin*, *dpp*, *dsh*, *ck1*, *apc*, *lrp5/6*, *shrp4*, *frizzled1/2/7*, and *wnt11*, Figure 6B) were up-regulated from LT to ES, and down-regulated from SL to WL, suggesting that these genes may be involved in function maintenance of larval segments in *U. unicinctus*, which was similar to the model of *Drosophila* and Annelid *Platynereis dumerilii* segments maintenance [13,19,32]. Meanwhile, 8 *NK* genes (*NK1*, *NK4*, *Lbx*, *Msx*, *Tlx*, *NK5*, *Dbx*, and *Vax*) of 44 homeotic genes showed significant difference (*p* < 0.05) from SL to WL, but only 1 *Hox* gene (*lox5*) and 1 *ParaHox* gene (*pdx*) showed significant difference (*p* < 0.05) (Figure 6C), indicating that these eight *NK* genes may also be involved in function maintenance of larval segments in *U. unicinctus*, like segment polarity genes. To date, research has shown that *NK1*, *NK4*, *Tlx*, *Msx*, and *Lbx* are expressed in a segment polarity-like pattern early in the development of the Onychophoran *Euperipatoides rowelli* [33] and the Annelid *P. dumerilii* [25]. Those results imply that *NK* genes may be involved in the formation of the body segment in both animals, although there are no functional studies on them in onychophorans and annelids currently. The above results proved that the segment polarity mechanism in *Drosophila* is conserved in echiurans, annelids, and arthropods, but the gap genes and pair-rule genes do not support a role in *U. unicinctus* segmentation. Furthermore, *NK* genes may be involved in the formation and maintenance of larval segments in *U. unicinctus*.

### 3.2. Key Genes and Pathways Involved in U. unicinctus Larval Segment Formation and Secondary Loss

As consistent with the trend of larval segment formation and secondary loss in the echiuran worm *U*. *unicinctus*, the expression levels of DEGs of profile 16 (Figure 4) were firstly increased and reached to top in ES and SL, and then decreased. Most of the segment formation-related genes can be found in this profile, such as *en*, *hh* and *wg*, the three major segment polarity genes. Moreover, *dsh* and *smo* were also enriched in this profile. The *dsh* gene encodes a protein that is an essential component of Wnt/β-catenin signal and it plays a key role in segment polarity in the early embryo of *Drosophila* [34,35]. The *smo* gene is also a segment polarity gene required for correct patterning of every segment in *Drosophila* and the Annelid *P. dumerilii*, which encodes a seven-pass membrane protein, a receptor for the Hedgehog signal [36,37]. The expression levels of DEGs in the profile 9 were expressed stably during the stages of LT to SL, and then decreased at the stage of WL. Similarly, many segment formation-related genes were enriched in this profile like *ptc* and *β-catenin*. These two genes were regarded as segment polarity genes in *Drosophila*, belonging to the Hedgehog and Wnt/β-catenin pathways, respectively [38,39]. Therefore, it was considered that DEGs of profile 16 and profile 9 were closely involved in larval segment formation and secondary loss in *U*. *unicinctus*.

To find new pathways from profile 16 and profile 9, the KEGG pathway annotation and enrichment were conducted. Finally, 8 key pathways (Table 4) were identified, out of which three (Hedgehog, Wnt, and Notch) pathways were known to be involved in the segment (or somite) formation in annelids, arthropods, or chordates [22]. Hedgehog signaling plays a central role in the development of most metazoans, which is originally identified as a mutation that causes a “segment polarity” phenotype in *Drosophila* [40]. Recent research indicates that the function of the Hedgehog pathway in “segment polarity” is conserved in other insects [20] or noninsect arthropods [41,42] and probably also in most annelids [13,43]. Wnt signaling is a highly conserved cellular communication system that regulates a wide range of developmental processes, including axis elongation and segmentation [44]. Studies of *wg*-*en* regulatory system in arthropods and annelids indicated that their delineation of segmental boundaries is an ancestral feature [13,45]. Delta/Notch signaling controls a wide spectrum of developmental processes, including body and leg segmentation in arthropods and segmentation clocks in vertebrates [12,46]. However, the other five pathways obtained in our study have not been confirmed to be associated with the segment formation, and the expression tendencies of the genes in these five pathways are consistent with the segment polarity genes in *U*. *unicinctus*. The TGF-β pathway can regulate diverse processes as cell proliferation, differentiation, motility, adhesion, organization, and programmed cell death [47]. The PI3K/Akt and AKT/mTOR pathways are critical for cellular proliferation, growth, survival, and mobility [48]. Dorso-ventral axis formation, during *Drosophila* embryogenesis, is required for the establishment of dorsoventral cell fates, determination of segmental identity, maintenance of amnioserosa and ventral neuroectodermal cells, germ band retraction, and production of cuticle [49]. MAPKs are serine-threonine protein kinases that play an important role in the regulation of many cellular processes including cell growth and proliferation, differentiation, and apoptosis [50]. In the future, it is necessary to conduct functional studies on whether these pathways participate in the formation and maintenance of segments in *U. unicinctus*.

### 3.3. Hedgehog Is a Conservative Gene for the Larval Segment Boundary Definition in U. unicinctus

Researchers show that the segmental subdivision of the *D. melanogaster* embryo along its anteroposterior axis is regulated by the interaction of a cascade of factors. The para-segmental boundaries are defined and maintained by the establishment of *hedgehog*, *engrailed*, and *wingless* expression domains and their mutual interaction. Loss of Hedgehog activity leads to the breakdown of segment boundaries [51]. Functional studies confirmed a conserved role for Hedgehog in the maintenance and patterning of segments in the Arthropod *Tribolium castaneum* [20] and the Annelid *Platynereis dumerilii* [19]. However, knowledge of its functional role in echiurans is still fragmentary. Our results of HH expressional profiles (Figure 7) suggested that HH may participate in the defining of the larval segment boundary in *U. unicinctus* and its function is evolutionarily conserved with annelids and arthropods.

## 4. Materials and Methods

### 4.1. Ethics Statement

The collection and handling of the *U. unicinctus* were performed in accordance with the Institutional Animal Care and Use Committee of the Ocean University of China (OUC-IACUC) and the local government on 15 June 2016.

### 4.2. Animals and Sampling

Adult *U. unicinctus* was collected from a coastal intertidal flat in Yantai, China. Mature sperms and ova were obtained by dissecting the nephridia (gonoducts) of male and female worms. Artificial insemination was conducted by mixing the sperms and ova with a ratio of 10:1 in filtered seawater (FSW). The fertilized eggs were reared in FSW (17 °C, pH 7.7, and salinity 30), and the hatched larvae were fed with single-cell algae (*Isochrysis galbana*, *Chlorella vulgaris*, *Chaetoceros muelleri*). The larvae at different stages were sampled and fixed in 4% paraformaldehyde for 15 h, and then dehydrated with serial methanol (25%, 50%, 75%, and 100%) and stored in 100% methanol at –30 °C for immunofluorescent histochemistry analysis. The larvae from four developmental stages, late-trochophore (LT, 25 days after hatching), early-segmentation larva (ES, 30 days after hatching), segmentation larva (SL, 35 days after hatching) and worm-shaped larva (WL, 42 days after hatching) were collected, frozen with liquid nitrogen immediately and then stored at −80 °C, respectively for total RNA extraction. Three biological replicates from each developmental stage were prepared.

### 4.3. RNA Extraction, RNA-Seq Library Construction, and Sequencing

Total RNA was extracted from each stored larval sample using Trizol (Invitrogen, Carlsbad, CA, USA) according to the manufacturer’s instructions, and treated with RNase-free DNase I (TaKaRa, Dalian, China) for 30 min at 37 °C to remove residual DNA. The concentration, purity, and integrity of the RNAs were assessed using NanoDrop 2000 (Thermo Scientific, Wilmington, DE, USA), and agarose gel electrophoresis were performed to assess the quality of total RNAs. Five μg total RNA per sample was used for RNA-Seq library preparation. The mRNA was isolated using oligo-dT beads (Qiagen, Hilden, Germany) and then broken into short fragments by adding fragmentation buffer. The first-strand cDNA was generated using random hexamer-primer with the short fragments as templates. The second-strand cDNA was synthesized using dNTPs, RNase H, and DNA polymerase I. The cDNA fragments were purified using a QiaQuick PCR extraction kit (Qiagen, Hilden, Germany). These purified fragments were washed with EB buffer for end reparation and poly(A) addition and then ligated to sequencing adapters. After agarose gel electrophoresis, the suitable fragments were selected as templates for the PCR amplification to construct the cDNA library. Finally, these libraries (total 12 libraries from 12 RNA samples) were respectively sequenced using Illumina HiSeq X Ten system (Illumina, San Diego, CA, USA) with the paired-end sequencing of 150 bp by Novogene Company (Beijing, China).

### 4.4. De Novo Assembly and Functional Annotation

The raw reads were firstly filtered to obtain high-quality sequences (clean reads) by removing the reads containing adapter sequences, ambiguous bases (‘N’ < 5%), and the low-quality reads with the Phred quality score < 20. All clean reads from the twelve libraries were jointly assembled into unigenes using Trinity software [52]. Finally, unigenes were annotated with NR, NT, Swissprot, KOG, Pfam, GO, and KO databases. The sequence direction of the unigenes was determined according to the best alignment results. When the results were conflicted among databases, the direction was determined consecutively by NR, NT, Swissprot, KOG, Pfam, KOG, and KO. When a unigene was not aligned to any database, ESTScan (http://myhits.isb-sib.ch/cgi-bin/estscan) was used to predict coding regions and determine sequence direction. GO annotation was performed by Blast2GO software (https://www.blast2go.com/). Functional classification of the unigenes was performed using WEGO software (http://wego.genomics.org.cn/).

### 4.5. Enrichment and Dynamic Expression Profile of Differentially Expressed Genes (DEGs)

The mapped fragments were normalized according to the expected number of fragments per kilobase of transcript sequence per million base pairs sequenced (FPKM) for each gene, which facilitated the comparison of transcript levels between samples. Differential expression genes (DEGs) between each of the larval stages (LT vs. ES, LT vs. SL, LT vs. WL, ES vs. SL, ES vs. WL, and SL vs. WL) were identified by the DEseq2 R package, and DEGs were determined as the FDR (False discovery rate) < 0.05 and |log2(foldchange)| ≥ 1. Go enrichment analysis of the DEGs was performed using GOSeq R package with the Wallenius non-central hypergeometric distribution model to adjust gene length bias in DEGs. KEGG pathway enrichment analysis of the DEGs was done using KOBAS [53] with the hypergeometric distribution model. The enrichment p-values were adjusted using the Benjamin and Hochberg method and significance was determined with adjusted *p* < 0.05. In addition, the expression patterns of DEGs during the larval development were determined with clusters generated by STEM [30].

### 4.6. Validation of RNA-Seq Data with Quantitative RT-PCR (qRT-PCR)

To examine the reliability of the transcriptome data, 8 differently expressed genes, *wnt11*, *smo*, *nk1*, *post2*, *abca3*, *nlk*, *rp-sae*, and *odd* were validated by qRT-PCR with the 12 RNA samples used for the transcriptional analysis. The cDNA was synthesized for each sample using Prime-ScriptTM RT reagent Kit (TaKaRa, Dalian, China) with the gene-specific primers (Table S2) designed using Primer Premier 5.0 according to their predicted CDS sequences. The amplifications were performed with SYBR Premix Ex Taq kit (TaKaRa, Dalian, China) in LightCycler 480 Real-Time PCR system. The PCR mixture consisted of 10 μL SYBR Premix Ex Taq II, 2 μL template cDNA, 2 μL forward primer (10 μM), 2 μL reverse primer (10 μM), and 4 μL ddH_2_O. The qRT-PCR condition was: denature at 95 °C for 30 s, followed by 39 cycles of 5 s at 95 °C, and 60 °C for 30 s. Each sample was run in 3 technical replicates. The relative expression levels were normalized to the reference gene *ATPase*, and expression ratios were calculated using the 2^–ΔΔ*C*t^ method. The experimental data were presented as the mean ± standard deviation from three samples with three parallel repetitions, and all qRT-PCR assays were validated in compliance with “the MIQE guidelines” [54]. Significant differences between means were tested using a one-way analysis of variance (ANOVA) followed by Tukey’s HSD test with SPSS software 18.0 (SPSS Inc., Chicago, IL, USA). The significance level was set at *p* < 0.05.

### 4.7. Immunofluorescence Histochemistry

The larvae samples were rehydrated in a gradient methanol series (100%, 75%, 50%, and 25%), and blocked with 3% bovine serum albumin (BSA) (Shanghai biotechnology Technology, Shanghai, China) diluted by PBT (pH 7.4). Subsequently, the samples were transferred into preimmune serum, or primary antibody (the specific anti-HH polyclonal antibody prepared with the HH recombinant protein detailed in Figure S4) diluted 1:200 in BSA and incubated overnight at 4 °C on a nutator. Afterward, the samples were rinsed in PBT for 2 h and incubated subsequently with secondary fluorochrome-conjugated antibodies (donkey anti-rabbit Alexa Fluor 488, Invitrogen, CA, USA) diluted 1:300 in PBT for 2 h. At last, the larvae were washed six times in PBT and incubated in PBT with 2.5% DAPI (Solarbio, Beijing, China) in the dark for 2 h to label cell nuclei. Negative controls were obtained by preimmune serum in order to check for antibody specificity. All the samples were analyzed with the confocal laser-scanning microscope Nikon A1RSi (Nikon, Tokyo, Japan). Drawings and final panels were designed using Adobe Photoshop (Adobe, San Jose, CA, USA).

## 5. Conclusions

Our study presented the first transcriptome analysis focusing on the gene expression profiles of the segment formation and secondary loss in *U. unicinctus*, in which 101 genes were identified involving in the segment formation as in *Drosophila* and annelids. Most of the segment polarity genes were conserved in echiurans, annelids, and arthropods, while the gap genes and pair-rule genes were not. Besides, *NK* genes may be involved in the formation and maintenance of larval segments in *U. unicinctus*. Moreover, eight key pathways were identified to be involved in *U. unicinctus* larval segment formation and secondary loss. We also verified that HH might participate in the defining of the larval segment boundary in *U. unicinctus* and its function is evolutionarily conserved with annelids and arthropods. This study provides a basic understanding of the molecular mechanism of larval segment formation in echiurans.

## Figures and Tables

**Table 1:** Overview of the sequencing reads from the twelve larval transcriptomic libraries of *U. unicinctus*.

Sample	Raw Reads	Clean Reads	Clean Bases	Error (%)	Q20 (%)	Q30 (%)	GC (%)
LT_1	44,405,794	43,232,040	6.48 Gb	0.02	95.41	89.03	46.93
LT_2	45,768,482	44,516,626	6.68 Gb	0.02	95.44	89.05	41.53
LT_3	51,280,946	49,850,242	7.48 Gb	0.01	97.44	93.49	45
ES_1	49,666,198	48,375,494	7.26 Gb	0.01	97.75	94.21	45.34
ES_2	46,441,732	45,203,342	6.78 Gb	0.01	97.81	94.3	47.12
ES_3	52,824,412	51,511,036	7.73 Gb	0.01	97.8	94.29	45.62
SL_1	50,346,424	48,909,398	7.34 Gb	0.01	97.83	94.34	47.75
SL_2	51,551,170	50,039,830	7.51 Gb	0.01	97.62	93.86	49.64
SL_3	65,816,438	63,885,972	9.58 Gb	0.01	97.71	94.1	46.84
WL_1	55,190,790	53,346,656	8 Gb	0.01	97.6	93.8	48.91
WL_2	50,393,708	48,640,910	7.3 Gb	0.01	97.58	93.78	48.88
WL_3	45,847,850	44,065,776	6.61 Gb	0.01	97.64	93.9	48.68

Q20/Q30: percentage of the bases with a quality value larger than 20 or 30.

**Table 2:** Length distribution of the assembled transcripts and unigenes from the *U. unicinctus* larval transcriptome.

	Min Length	Mean Length	Median Length	Max Length	N50	N90	Total Nucleotides
Transcripts	201	640	291	19,744	1206	245	355,841,139
Unigenes	201	1125	654	19,744	1836	464	273,911,152

N50/N90: the shortest sequence length at 50%/90% of the total length of the spliced transcripts.

**Table 3:** *U. unicinctus* larval transcriptome unigenes annotations against the public databases.

Item	NR	NT	KO	SwissProt	Pfam	GO	KOG	In All Databases	At Least One Database
No. of genes	122,354	22,899	33,027	94,085	109,847	111,479	71,895	7704	149,488
Percentage	50.27%	9.4%	13.57%	38.65%	45.13%	45.8%	29.54%	3.16%	61.42%

**Table 4:** Key pathways significantly enriched from profile 9 and profile 16 in *U. unicinctus* larval transcriptome.

Pathway	Pathway ID	*p*-Value (profile9)	*p*-Value (profile16)
Hedgehog signaling pathway	ko04341	0.04767	0.00608
Wnt signaling pathway	ko04310	0.03753	0.00201
TGF-beta signaling pathway	ko04350	0.03045	0.01209
PI3K-AKT signaling pathway	ko04068	―	0.00428
mTOR signaling pathway	ko04150	―	0.00012
MAPK signaling pathway	ko04010	―	0.0085
Notch signaling pathway	ko04330	0.04927	―
Dorso-ventral axis formation	ko04320	0.00831	0.00909

*“―”* means not significantly enriched (*p* > 0.05).

## Supplementary Materials

Supplementary materials can be found at https://www.mdpi.com/1422-0067/20/8/1806/s1. Table S1. Expression trends of the 101 gene homologs involved in segment formation in *Urechis unicinctus* transcriptome; Table S2. Primer sequences of the DEGs for qRT-PCR; Figure S1. PCA analysis of *U. unicinctus* larval samples used for transcriptional analysis; Figure S2. Functional annotation of all unigenes in *U. unicinctus* larval transcriptome; Figure S3. Immunofluorescence assay with preimmune serum as a negative control in *U. unicinctus* larvae during the segment formation and secondary loss; Figure S4. Expression and purification of *U. unicinctus* Hedgehog recombinant protein analyzed by SDS-PAGE and specificity analysis of the anti-Hedgehog polyclonal antibody detected by Western blotting.

## Abbreviations

LT	late-trochophore
ES	early-segmentation larva
SL	segmentation larva
WL	worm-shaped larva
DEG	Differentially expressed gene
FDR	False discovery rate
STEM	Short time-series expression miner
qRT-PCR	Quantitative real-time PCR
FPKM	Fragments per kilobase of transcript sequence per million base pairs sequenced
HH	Hedgehog protein
